# Mathematical Logic in the Human Brain: Syntax

**DOI:** 10.1371/journal.pone.0005599

**Published:** 2009-05-28

**Authors:** Roland Friedrich, Angela D. Friederici

**Affiliations:** Max Planck Institute for Human Cognitive and Brain Sciences, Leipzig, Germany; University of Groningen, Netherlands

## Abstract

Theory predicts a close structural relation of formal languages with natural languages. Both share the aspect of an underlying grammar which either generates (hierarchically) structured expressions or allows us to decide whether a sentence is syntactically correct or not. The advantage of rule-based communication is commonly believed to be its efficiency and effectiveness. A particularly important class of formal languages are those underlying the mathematical syntax. Here we provide brain-imaging evidence that the syntactic processing of abstract mathematical formulae, written in a first order language, is, indeed efficient and effective as a rule-based generation and decision process. However, it is remarkable, that the neural network involved, consisting of intraparietal and prefrontal regions, only involves Broca's area in a surprisingly selective way. This seems to imply that despite structural analogies of common and current formal languages, at the neural level, mathematics and natural language are processed differently, in principal.

## Introduction

In a remarkable but controversially discussed paper [1], Hauser, Chomsky and Fitch made the claim that one of the distinctive features that separates humans from non-human primates is the ability to process hierarchical structures as found in (natural) grammars. In line with this theory, it was demonstrated that humans were able to easily learn an artificial Finite State Grammar (FSG) (i.e., “flat structures”) and also a Phrase Structure Grammar (PSG) (i.e., “hierarchical structures”) whereas monkeys were only able to learn the FSG, and not the PSG [2]. For humans it was subsequently shown [3] that the processing of PSG involved the Broca's area (a fundamental region of human language processing) in the left hemisphere in addition to a phylogenetically older brain region able to deal with the FSG. This led to the conclusion that processing hierarchical structures, as arising in grammars, draws on a particular circumscribed brain area in humans.

As closer examination reveals that examples of hierarchically organised data or information are abundant in everyday life. A familiar form of it is already apparent in simple equations or algebraic expressions, even if one usually does not perceive them as such when dealing with them. However, what they have in common with (natural) languages is the fact that the formation of the hierarchy in mathematical expressions is not arbitrary, but obeys strict rules, rules which not only apply to their generation but also to their interpretation (e.g., calculation). These rules, however, do not necessarily follow the principles of natural languages.

Here, we looked at the neural base of mathematics from this novel perspective, with Mathematical Logic as the obvious “language-mathematics interface”. We also added a new aspect, namely by also including the case where the processor (i.e., the human brain) encounters an “almost” well-defined structure, which is tantamount to error detection during interpretation.

The question of what the sources of mathematical thinking at the neural level might be has already been raised [4]–[7]. The focus, however, has almost exclusively been on the number sense, that is on the capabilities of the human brain to do either exact or approximate arithmetic or simple algebraic calculations. It was found that the intraparietal sulcus (IPS) was systematically activated in all number tasks and therefore it was concluded that it can host a central amodal representation of quantity.

However, in light of the above discussion and also from a modern standpoint which focuses on structures, objects and relations, the “number” approach not only inevitably falls short in recognising the essence of the cognitive roots of mathematics but also in relating it to other fundamental cognitive domains, such as language, for example.

**Figure 1:**
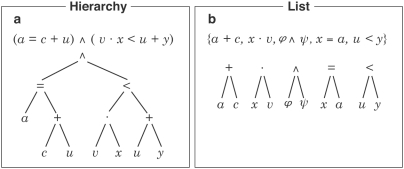
Illustration of the underlying binary tree structures for the various expressions used in the (a) hierarchical syntactic condition, and (b) list (non-hierarchical) condition. The grammatical/generative part of the syntax starts with an alphabet, out of which termini and formulae are (recursively) built, thereby yielding arbitrary hierarchical expressions. For assumed processing steps see (Supplemental Information: Figure S1).

Therefore, we designed an experiment using functional magnetic imaging (fMRI) to investigate the syntactic processing of abstract mathematical formulae and termini, written in a standard first-order language. The stimuli items used (see Figure 1(a) and (b)) represented either first-order hierarchical formulae or termini in a list. All expressions were either syntactically correct or incorrect, but were always without any semantic meaning.

We predicted to find activation beyond those areas known to support number processing i.e., the intraparietal region.

Under the hypothesis that first-order languages, which are by definition formal languages, share a neural representation with other formal languages, we expect activation in the left inferior frontal gyrus (IFG), in particular Broca's area, as this region has been found to activate during the processing of syntactic hierarchical structures in artificial grammars (e.g., [3], [8]) and in natural languages [9]–[11]. Further, we expected activation in the prefrontal cortex as a result of error detection [12].

## Results

### Behavioural results

As responses were given only after stimulus presentation in a delayed mode, only the percentage of correct responses and no reaction time data could be analysed. Overall, performance data from the present fMRI experiment showed that the 24 participants answered correctly on an average of 88% (standard deviation (SD) of 0.12) on the 50 hierarchical items compared to 96% (SD 0.05) on the 50 non-hierarchical ones. This difference was significant for the two-tailed paired *t*-test at the 5% level.

A subgroup of 12 participants, however, showed no significant difference in performance with respect to the two types of problems. For this sub-group, the mean correct answers for hierarchical formulae was 94% (SD 0.068) and 95% (SD 0.062) for the lists, for the two-tailed paired *t*-test at the 5% level. To rule out the possibility that the difference in performance would have an effect on the pattern of brain activation at the group level, we conducted two further statistical tests. A two-sample *t*-test, comparing the fMRI activation for the above 12 people with the other 12 yielded the result that there was no significant difference (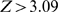, 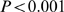 uncorrected). Additionally, we conducted a parametric test for all 24 participants with the ratios of correct answers for both types as covariates. Again, there was no effect (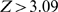, 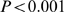 uncorrected).

Therefore, all results are reported for the entire group of 24 participants. On average these 24 participants performed correctly with an average of 86% (SD 0.12) on the correct hierarchical items and 89% (SD 0.13) on the incorrect hierarchical items. This difference was not significant for the two-tailed paired *t*-test at the 5% level. Also, participants performed correctly with an average of 98% (SD 0.02) on the correct list items and 94% (SD 0.08) on the incorrect list items. This difference was significant for the two-tailed paired *t*-test at the 5% level.

### fMRI results

**Figure 2:**
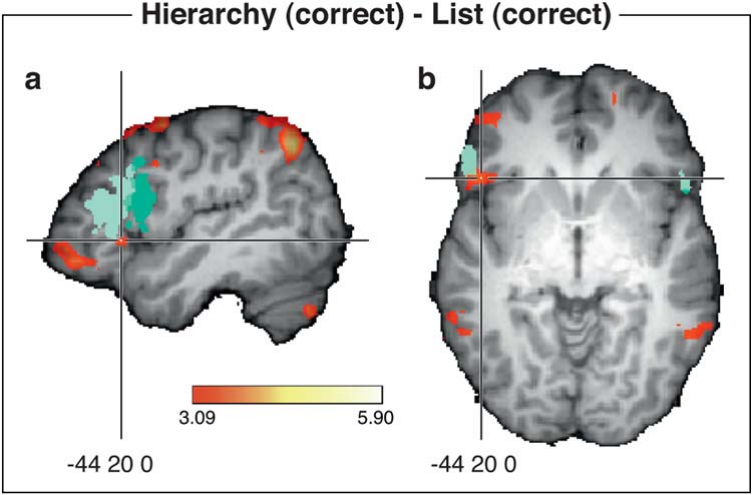
The figure shows activation for correct hierarchical syntactic formulae relative to a list of correct flat syntactical sequences. FMRI data are mapped onto a reference brain (single subject), where areas differing significantly in activation are coloured red to yellow and correspond to values with 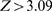 (uncorrected). The cross hair is placed at (−44, 20, 0) in the Talairach co-ordinate system. Views: (a) sagittal 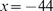 and (b) axial 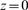. The entire region marked in green in (a) and (b), corresponds to the cytoarchitectonically defined Broca's area with a probability of at least 50% according to [13]. Light green corresponds to Brodmann area BA 45, and dark green to BA 44.

When comparing the fMRI data of the entire group for the processing of correct hierarchical structures to those of correct flat structures (see Figure 2 and Table 1), bilateral activation was found in the inferior parietal lobe which in the right hemisphere extended to the occipital lobe, moreover, bilateral activation was observed in the middle temporal gyri. In addition, the comparison revealed bilateral activation in the middle frontal gyri (BA 6) and in (BA 10), and also in the left IFG (BA 45/46/47). Note the non-overlap of the present IFG activation with the cytoarchitectonically defined Broca's area ([13], for details see Figure 2).

**Table 1:** Hierarchical correct vs. List correct.

AREA	Talairach co-ordinates						
	left			right			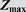
mFG, BA 10	−38	52	−3	–	–	–	**4.20**
mFG, BA 10	–	–	–	31	49	−9	**3.43**
IFG, BA 45	−47	19	6	–	–	–	**3.34**
IFG, BA 47	−38	37	−3	–	–	–	**3.92**
MFG, BA 6	−41	10	51	–	–	–	**3.94**
MFG, BA 6	–	–	–	34	16	45	**3.87**
SFG, BA 6	−23	19	60	–	–	–	**3.63**
MTG, BA 21	−65	−53	3	–	–	–	**3.52**
MTG, BA 22	–	–	–	55	−50	0	**4.14**
AG, BA 39	–	–	–	37	−74	33	**4.21**
Inf. parietal lobule, BA 40	−53	−41	45	–	–	–	**4.39**
Inf. parietal lobule, BA 40	–	–	–	43	−47	42	**4.62**
Precuneus, BA 7	−5	−65	45	–	–	–	**4.27**
Cuneus, BA 18	–	–	–	13	−77	−18	**3.35**

Activation maxima (uncorrected) of the contrast: “hierarchical correct vs. list correct”. Abbreviations: AG: angular gyrus, BA: Brodmann area, IFG: inferior frontal gyrus, mFG: medial frontal gyrus, MFG: middle frontal gyrus, MTG: middle temporal gyrus, SFG: superior frontal gyrus.

**Figure 3:**
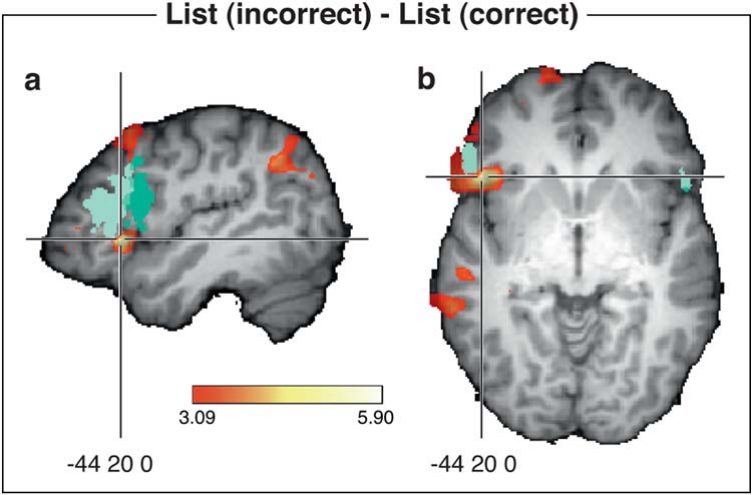
The figure shows activation for incorrect flat sequences vs. correct flat sequences. FMRI data are mapped onto a reference brain (single subject), where areas differing significantly in activation are coloured red to yellow and correspond to values with 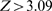 (uncorrected). The cross hair is placed at (−44, 20, 0) in the Talairach co-ordinate system. Views: (a) sagittal 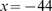 and (b) axial 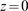. The entire region marked in green in (a) and (b), corresponds to the cytoarchitectonically defined Broca's area with a probability of at least 50% according [13]. Light green corresponds to Brodmann area BA 45, and dark green to BA 44.

When we compared the incorrect list items to the correct ones for the entire group (see Figure 3 and Table 2), we found bilateral activation in the angular gyrus, the middle frontal gyrus (BA 6) and the IFG (BA 47). In addition, left (BA 10) and left (BA 22) as well as the dorsolateral prefrontal region (BA 8) showed a significant activation.

**Table 2:** Flat incorrect vs. Flat correct.

AREA	Talairach co-ordinates						
	left			right			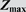
mFG, BA 10	−14	67	−3	–	–	–	**3.90**
mFG, BA 8	−5	40	36	–	–	–	**4.09**
IFG, BA 47	−44	19	0	–	–	–	**5.91**
IFG, BA 47	–	–	–	22	10	−15	**3.56**
SFG, BA 6	−11	19	63	–	–	–	**4.64**
MFG, BA 6	–	–	–	34	7	48	**3.92**
AG, BA 39	–	–	–	37	−59	36	**4.65**
AG, BA 39	−38	−56	33	–	–	–	**4.19**
Cingulate G, BA 31	−5	−35	39	–	–	–	**3.96**
MTG, BA 22	−53	−41	3	–	–	–	**4.39**

Activation maxima (uncorrected) of the contrast: “flat incorrect vs. flat correct” expressions. Abbreviations: AG: angular gyrus, BA: Brodmann area, IFG: inferior frontal gyrus, G: gyrus, mFG: medial frontal gyrus, MFG: middle frontal gyrus, MTG: middle temporal gyrus, SFG superior frontal gyrus.

Further, the comparison of the correct hierarchical condition with the baseline condition and also the comparison of the correct list condition with the baseline condition, revealed no overlap with the cytoarchitectonically defined Broca's area ([13], for details see Figures S3, S4 and S5 in the Supporting Information).

## Discussion

At a macroscopic level the present experiment found significant inferior frontal, middle frontal and parietal activation for the processing of the syntax of first order logic (“mathematical syntax”) of correct hierarchical structures compared to correct non-hierarchical structures as represented by the mathematical expressions used.

To understand the activation observed, it is necessary to identify the main processing modules needed to accomplish the given task successfully. One would expect processing to rely on a tangible neural network involving several modules which interchange information and interact as time elapses. The modules should grant visual decoding (“reading”) of the visually presented stimuli, allow mental transformations of the formulae/termini (visuospatial working memory), retrieval and application of the rules underlying the proper generation of the syntax of first-order logic, and finally preparation of the response.

The first processing step (i.e., reading) is necessary for both conditions (formulae and termini) and, therefore should not show up in a direct comparison between conditions, as was indeed the case. The observed bilateral parietal activation surrounding the entire intraparietal sulcus (IPS) replicates part of a neural network previously found in (arithmetic or algebraic) calculation tasks [4]–[7] and is proposed to host a central amodal representation of quantity. However, the combined bilateral activation of the IPS and the cortices along posterior parts of the superior frontal sulcus (BA 6), as in the present experiment, has been shown to form a brain system on which visuospatial working memory relies [14]. This is consistent with our expectations, as the visual memory load is much higher for formulae with their long-range dependencies compared to the single items in the list, which correspond to local dependencies.

It is assumed ([15] for a review) that the neural basis underlying (long-term) memory comprises the medial temporal lobe (MTL) and also the prefrontal cortex (PFC). These two brain regions have to interact with each other to either encode perceived information or to retrieve it. Specifically, it is known that (BA 37) in the MTL participates in the analysis of visual forms such as characters and the representation of objects.

Further, the dorsolateral part of the prefrontal cortex, (DLPFC, BA 46 and BA 9) is assumed to be engaged in organisation of material to be remembered in encoding interactions, and, during retrieval interactions, in monitoring and verifying retrieved information. This is in line with the activation observed, as a model of processing hierarchical formulae that assumes both more memory resources for more material to be remembered and also more verification steps at each node in the hierarchical compared to the list condition.

The observation of activation in the left IFG (in BA 45/47) as a function of the processing of the syntactic hierarchy in abstract formulae is novel for two reasons. First, activation in the left IFG, as observed during arithmetic tasks in previous studies, which are notably by definition semantic and not syntactic, has been attributed to general working memory [15] or to verbal aspects of mental calculations [6]. The present activation, however, can be considered to be specific to the processing of mathematical syntax within the domain of mathematics, as it results from a comparison between a string representing a list structure and a hierarchical one, with the hierarchy introducing an additional degree of freedom.

Second, outside the domain of arithmetic calculations, processing activation in the left IFG has been found in a number of studies on language, with different sub-regions reflecting different aspects of language processing. Activation in the more posterior portion of the IFG ( i.e., in BA 44 and posterior portion of 45) has been observed for the processing of hierarchical sentence structures as compared to flat structures in German [9], [10], Hebrew [11] and for artificial grammars [3], [8]. Thus it appears that the present first-order language does not recruit the same areas as natural languages when dealing with hierarchical structures. The present finding is in line with the view that syntactic rules of a natural grammar recruit different brain regions than rules that do not follow the principles of natural languages [16]. The present data indicate that the processing of hierarchical versus non-hierarchical structured mathematical formulae is based on a region in the inferior frontal cortex, namely in the ventral portion of BA 45/47 which is located more anteriorly and more ventrally than the brain region reported for the processing of hierarchical versus non-hierarchical structures in language (e.g. [3]). Activation in BA 45/47, a region that has rather been found for controlled semantic processes such as categorisation and relatedness judgement [17]. The most anterior and ventral portion for the IFG (i.e., in BA 47 proper) has been reported to activate during sequences learning in an artificial grammar [18] and, moreover, has been implicated in general intelligence, as it was seen to be activated in a number of tasks in which sequences structured by analogies had to be judged for coherence [19].

Thus, the activation in the anterior prefrontal cortex, i.e. BA 45 and BA 47 has been observed, in the context of studies investigating “semantic processing” in language [20] and also with more general concepts such as “general intelligence” [19] or “deductive reasoning” [21]. BA 47 comes into play when processing novel or complex relations in structured sequences. In the present experiment, we can interpret this activation as coming from deductive mental operations during the application of the syntactic rules underlying the formation of hierarchical formulae in the process of the verification of whether a given string of symbols represents a correct formula or not. Again, according to our processing model, hierarchically structured formulae require more such “basic inference steps” when compared to lists. Against the background of these data, the recruitment of BA45/47 in the processing of mathematical hierarchies in the present study suggests that even in people with mathematical training, the brain still considers hierarchically structured mathematical formulae to be complex sequences.

The comparison of incorrect versus correct list items revealed significant activations in the left frontopolar cortex (FPC), bilaterally in the ventrolateral prefrontal cortex (VLPFC; BA 47), the anterior cingulate cortex (ACC; BA 32/8), the bilateral angular gyrus (BA 39), the bilateral middle frontal gyrus (BA6) and the left medial temporal lobe (BA 22). The number of activation foci exceeded by far those found [12] for the processing of incorrect, compared to correct, arithmetic equations with either two or three operands, but identically revealed the involvement of the VLPFC. The combined activation of ACC, medial PFC and the angular gyri suggests that once subjects detect an error, they check the expression again to minimise the uncertainty coming from the possibility that it is not the expression that is incorrect but rather that they have made an error [22], [23]. This is remarkable, inasmuch as such an uncertainty is by definition not inherent in the well-defined rules that generate the expressions, and for which reasons a “classical automaton” would a priori unambiguously judge the strings of symbols. Humans, in contrast, seem to add redundancy (checks) with the aim of error detection.

The additional comparisons “hierarchy vs. list” and “correct vs. incorrect” (see Supporting Information, Tables S1 and S2) further showed no effect in the prefrontal cortex (PFC) either in its anterior nor in its ventrolateral part. On the other hand, the activity in the medial PFC significantly correlated with the occurrence of incorrect expressions, which once more [22], [23] shows its critical participation in the resolution of uncertainty and its role as a trigger for action changes induced by negative stimuli (error). Finally, the combined contrasts calculated suggest that it takes almost the same amount of prefrontal involvement to process a correct hierarchical structure as it takes to deal with a “simple error” as provided in the list case. This is far-reaching, inasmuch as it hints at an explanation at the neural level of why a rule-based system proves to be advantageous, as such a system allows us to deal efficiently both with complex and with erroneous structures.

### Conclusion

When taking into account the brain activations across different studies, the combined data suggest a functional differentiation between more posterior and more anterior portions of the IFG, with more anterior portions being recruited the more complex the relation between elements in a structured sequence are. This assumption of such a graduation from more posterior to anterior IFG receives support from two perspectives, these being evolutionary neuroanatomy [24] and functional brain imaging [25], [26]. According to these perspectives, the frontal cortex can be viewed as being graduated from the precentral gyrus (BA 6) towards the posterior portion of BrocaÕs area (BA 44) and the more anterior portions (BA 45/47). Some neuroanatomical views hold that the younger an area is with respect to its evolutionary status, the more anterior it is located [24], and functional imaging data indicate that the more complex an action sequence and the abstract relational hierarchy, the more anterior the activation in the IFG [25]–[27].

Finally, the present neuroimaging data suggests that a formal ruled-based generation and decision process as in the form of a calculus is effective because it strives for an optimal balance between data compression and reliability, implemented at the neural level. This in turn permits humans to communicate complexly structured information and to phrase problems more easily in face of the limits of the human processing system.

## Materials and Methods

### Subjects

Twenty-four participants gave their informed consent, after having read and signed the guidelines set out for fMRI studies at the Max Planck Institute for Human Cognitive and Brain Sciences. Specifically, we had 24 healthy, right-handed subjects (8 female, 16 male), who were German native speakers with normal or corrected to normal vision. The age range was from 21 to 31 years of age, (mean: 25.9 years, SD 2.6). Almost all participants were university students and all were part of the Institute's database of regular and general fMRI subjects.

### Stimuli

We based our specific first-order language on an alphabet consisting of: variables: 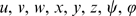; logical symbols (and, or): ∧,∨; equality:  = ; two types of left-right parenthesis: (,), {,}; a semicolon: , ; a binary relation symbol: <; two binary function symbols (plus, multiplication): +,· and constants: 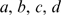.

The set of variables and constants was chosen randomly, whereas the selection of the other symbols followed more specific rules. The two variables denoted by Greek letters 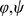 exclusively stood for formulae and were only used in the lists. This was necessary in order to use the conjunction and disjunction symbols to form non-trivial three letter strings (e.g., 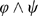).

Out of the symbols we built first-order formulae that were either syntactically correct or incorrect. The errors were violations of the well-defined building rules for terms and formulae in logic, and not just simple misprints.

An item for the visual presentation either corresponded to an entire formula, an entire list or the baseline picture. So, e.g.
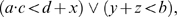
corresponded to one formula item and was presented as a whole, i.e., the complete above expression was visible at once on the screen. Analogously for the lists, e.g.
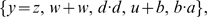
corresponded to one list item and was presented as a whole, i.e., the complete above list was visible at once on the screen, and not presented symbol by symbol.

There were 25 syntactically correct formula items (e.g. as the formula above), 25 incorrect formula items and correspondingly 25 correct list items (e.g. as the list above) and 25 incorrect list items, i.e. a total of 100 items (50 formulae and 50 lists) to be judged for their grammatical content.

The stimuli represented for the formulae either 1,2,3-binary-trees, i.e. trees with one node at the top, two at the second level and three at the third level, or a list consisting of five simple 1-trees, i.e., a “hedge” (cf. Fig. 1).

Stimuli of the following four types (1,2,0111;1,2,1011;1,2,1101;1,2,1110) were provided to ensure that subjects could not use the same reading strategy during the experiment.

For the baseline image, a row of white-greyish circles was used on a very dark grey background.

### fMRI Acquisition

The software packages used were LIPSIA [28] for the data analysis and PRESENTATION (Neurobehavioral Systems) for the visual presentation of the stimulus material. The study was conducted on a 3T BRUKER scanner (Medspec S300, Bruker, Ettlingen).

For registration purposes, two sets of two-dimensional anatomical images were acquired for each participant immediately prior to the functional imaging. An MDEFT and an EPI-T1 sequence were used. T1-weighted MDEFT images were obtained, with a non slice-selective inversion pulse followed by a single excitation of each slice. Anatomical images were positioned parallel to AC-PC.

The functional MRI were as follows; Axial slices: TR = 2 s, TE = 30 ms, alpha = 90°, 29 slices (29×4 mm = 11.6 cm, whole brain), 4 mm slice thickness (no gap), voxel volume: 3×3×4 mm^3^, 64×64 matrix, 19.2 cm FOV. There were 25 stimuli per condition (4 conditions+nullevent), presented with SOA = 7 s, with a total stimulation time of 27 minutes (25×5×13 seconds).

### fMRI Analysis

The data processing was performed using the software package LIPSIA [28]. This software package contains tools for pre-processing, co-registration, statistical evaluation, and visualisation of fMRI data. Pre-processing was carried out as follows: Functional data were motion-corrected using a matching metric based on linear correlation. To correct for the temporal offset between the slices acquired in one scan, a cubic spline-interpolation was applied. A temporal highpass filter with a cut-off frequency of 1/72 Hz was used for baseline correction of the signal and a spatial Gaussian filter with 6 mm FWHM was applied. The increased auto-correlation caused by the filtering was taken into account during statistical calculation by an adjustment of the degrees of freedom.

Subsequently co-registration of data was carried out. To align the functional slices with a 3D stereotactic co-ordinate reference system, a rigid linear registration with six degrees of freedom (3 rotational, 3 translational) was performed. The rotational and translational parameters were acquired on the basis of the MDEFT and EPI-T1 slices to achieve an optimal match between these slices and the individual 3D reference data set. This 3D reference data set was acquired for each subject during a previous scanning session. The MDEFT volume data set with 160 slices and 1 mm slice thickness was standardised to the Talairach stereotactic space [29]. The rotational and translational parameters were subsequently transformed by linear scaling to a standard size. The resulting parameters were then used to transform the functional slices using trilinear interpolation so that the resulting functional slices were aligned with the stereotactic co-ordinate system. This linear normalisation process was improved by a subsequent processing step that performed an additional non-linear normalisation.

The statistical evaluation was based on a least-squares estimation using the general linear model for serially auto-correlated observations. The design matrix was generated with a synthetic haemodynamic response function and its first and second derivative. The model equation, including the observation data, the design matrix and the error term, was convoluted with a Gaussian kernel of dispersion of 4 s FWHM to deal with the temporal auto-correlation. Afterwards, contrast-images (i.e., estimates of the raw-score differences between the specified conditions) were calculated for each subject. Each individual functional data-set was aligned with the standard stereotactic reference space, so that a group analysis based on the contrast-images could be performed.

The individual contrast-images were first masked and the individual and masked contrast-images were then entered into a second-level random effects analysis (one-sample *t*-test). Subsequently, *t*-values were transformed into *Z*-scores. A group analysis was performed by averaging individual *Z*-maps and multiplying each *Z*-value with the square root of the number of subjects in the experiment. Only regions with *Z*-score greater than 3.09 (uncorrected) and at least 8 contingent voxels were considered.

### Procedure

The experiment was devised as a reading experiment. The 125 stimuli items (50 stimuli of hierarchical type, 50 stimuli of list type and 25 baseline stimuli) were presented as a whole to the participants, in a fully randomised order. The presentation of hierarchical, flat and baseline conditions were intermixed. A stimulus item, e.g. a formula, was visible as a whole for a fixed period of 7600 ms on the screen. Randomisation was done using the random number generator of the computer programme “Presentation”, and was done for each subject separately. There was one run per participant with no repetition of formulae or list items, but the baseline item was always the same. The subjects' task was to judge the syntactic correctness of each of the formula or list items shown (for examples and assumed processing steps underlying the judgement in the different conditions see the Supporting Information Text S1, including Figures S1 and S2).

The response had to be given for each stimulus by the participant after the stimulus item disappeared from the screen and a new screen indicated that the answer had to be given. The participant had 1700 ms to press the respective button, i.e., one for correct and one for incorrect. No feedback was given after the button press. For the baseline condition no answer was required. (For a schematic description of the experiment, see Supporting Information, Figure S6)

All presentation material, including the visibility of the stimuli, was previously tested in the scanner. All participants were carefully instructed before the actual test and also had a training session with a sample of similar stimuli presented on a laptop and with a button press device.

## Supporting Information

Text S1 (0.02 MB DOC)

Figure S1 Assumed processing steps required to check the syntax of the hierarchical expression.
(0.73 MB EPS)

Figure S2 Assumed processing steps required to check the syntax of the list of expressions.
(0.73 MB EPS)

Figure S3 Broca's area (BA 44/45) (blue, green, lilac) and activations from contrast “hierarchy correct-baseline”. FMRI data are mapped onto a reference brain (single subject), where areas differing significantly in activation are coloured red to yellow and correspond to values with Z>3.09 (uncorrected). The cross hair is placed at (−44, 37, 1) in the Talairach co-ordinate system. Views: coronal y = 37, sagittal x = −44 and axial z = 1. The region marked in green, blue and lilac corresponds to the cytoarchitectonically defined Broca's area with a probability of at least 50% according to [13]. Green: BA 45, blue: BA 44, lilac: intersection of the two.
(0.09 MB TIF)

Figure S4 Broca's area (BA 44/45) (blue, green, lilac) and activations from contrast “list correct-baseline”. FMRI data are mapped onto a reference brain (single subject), where areas differing significantly in activation are coloured red to yellow and correspond to values with Z>3.09 (uncorrected). The cross hair is placed at (−44, 37, 1) in the Talairach co-ordinate system. Views: coronal y = 37, sagittal x = −44 and axial z = 1. The region marked in green, blue and lilac corresponds to the cytoarchitectonically defined Broca's area with a probability of at least 50% according to [13]. Green: BA 45, blue: BA 44, lilac: intersection of the two.
(0.09 MB TIF)

Figure S5 Broca's area is outlined in blue, and regions of activations with Z>3.09, are outlined in white for “hierarchy correct-list correct”, in yellow for “hierarchy correct-baseline” and in red for “list correct-baseline”. The cross hair is placed at (−50, 31, 24) in the Talairach co-ordinate system. Views: coronal y = 31, sagittal x = −50 and axial z = 24. The region marked in blue corresponds to the cytoarchitectonically defined Broca's area (50% according to [13])
(0.09 MB TIF)

Figure S6 Schematic illustration (not to scale) of the sequence of screen contents with the respective duration of each phase, of the fMRI experiment. (isi = inter stimulus interval)
(0.33 MB EPS)

Table S1 (0.01 MB DOC)

Table S2 (0.01 MB DOC)
