# Supplementary material for: Mathematical Logic in the Human Brain: Syntax
Source: PLoS One. 2009 May 28;4(5):e5599. doi: 10.1371/journal.pone.0005599 (PMC2685028; doi:10.1371/journal.pone.0005599)
Supplement: Text S1 — (0.02 MB DOC) [file pone.0005599.s001.doc]

# **Supporting Information**

**Processing Steps for Different Formula Types**

**Hierarchical Formula**

The steps that would be required to check the syntax of the expression in Supporting Information Figure S1, shall roughly be outlined in the following. The circles enclose the vertices of the tree with at least two edges ending or starting at it, and are enumerated from top to bottom by the numbers one to six.

Note, that we wrote the binary functions symbols, as in every day use, between the arguments, instead of in-front of them and omitted additional parentheses. Let us suppose that the person starts at the symbol  between the two parts in parentheses. We describe now the checking of the syntax.

**1. node: ** check if the left and right side are formulae, go left

**2. node: =**  check if left and right side are termini

on the left a, a constant, → ok,

go right

**4. node: +** binary function → arguments have to be variables or constants

c constant, → ok,
 u variable, → ok; hence a term

→ left of ∧ is a formula, go up and to the right of 1. node.

**3. node: =** check if left and right side are termini, go left

**5. node:** ▪ binary function → arguments have to be variables or constants

v variable, → ok,

x variable, → ok; hence a term

go to the right of 3. node.

**6. node +** binary function symbol: arguments have to be variables or constants

u variable, → ok,

y variable, → ok; hence a term

→ right of  is a formula, so the whole expression is syntactically correct

We note, that if at one place a syntactic error would have occurred, the complete expression would have been syntactically incorrect, and we would have immediately stopped to check further.

**Non-hierarchical Formula (list)**

The steps that would be required to check the syntax of the expression in Figure S2 shall, be outlined in the following.

Note, that we wrote the binary functions symbols, as in every day use, between the arguments, instead of, in-front of them .

Let us suppose that the person starts reading the list from left to right. The various items in the list are separated by a semicolon and represent the smallest syntactically meaningful non-trivial unit, namely a one tree. We describe now the checking of the syntax.

**1. item: +** binary function → arguments have to be variables or constants

a constant,→ok

c constant, → ok; hence a term, check next item

**2. item:** ▪ binary function → arguments have to be variables or constants

x variable, → ok,

v variable, → ok; hence a term, check next item

**3. item: ∧** conjuntion symbol: check if the left and right side are formulae

φ symbol for a formula, → ok,

ψ symbol for a formula, → ok;

→ right and left of ∧ stands a formula-symbol , so the whole expression is syntactically correct, check next item

**4. item: =**  equality: check if the left and right side are termini

x variable, → ok,

a constant, → ok; hence a formula, check next item

**5. item: **  binary relation: check if the left and right side are termini

u variable, → ok,

y variable, → ok; hence a formula, checking of the list is terminated

We note, that if at one place a syntactic error would have occurred, we would have immediately stopped to check further the list, as the rule was, that the list is incorrect if it contains a syntactically incorrect expression.
