# Supplementary figures and images for: Mathematical Logic in the Human Brain: Syntax
Source: PLoS One. 2009 May 28;4(5):e5599. doi: 10.1371/journal.pone.0005599 (PMC2685028; doi:10.1371/journal.pone.0005599)

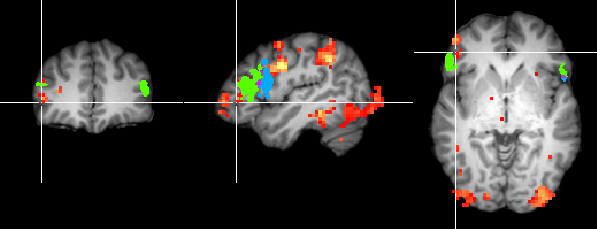

Supplement: Figure S3 — Broca's area (BA 44/45) (blue, green, lilac) and activations from contrast “hierarchy correct-baseline”. FMRI data are mapped onto a reference brain (single subject), where areas differing significantly in activation are coloured red to yellow and correspond to values with Z>3.09 (uncorrected). The cross hair is placed at (−44, 37, 1) in the Talairach co-ordinate system. Views: coronal y = 37, sagittal x = −44 and axial z = 1. The region marked in green, blue and lilac corresponds to the cytoarchitectonically defined Broca's area with a probability of at least 50% according to [13]. Green: BA 45, blue: BA 44, lilac: intersection of the two. (0.09 MB TIF) [file pone.0005599.s004.tif]

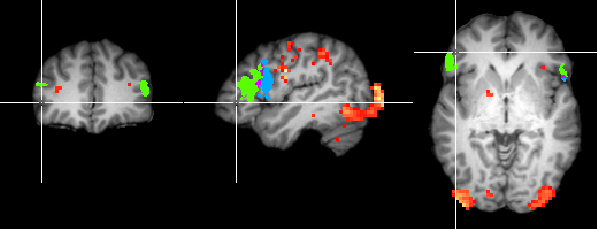

Supplement: Figure S4 — Broca's area (BA 44/45) (blue, green, lilac) and activations from contrast “list correct-baseline”. FMRI data are mapped onto a reference brain (single subject), where areas differing significantly in activation are coloured red to yellow and correspond to values with Z>3.09 (uncorrected). The cross hair is placed at (−44, 37, 1) in the Talairach co-ordinate system. Views: coronal y = 37, sagittal x = −44 and axial z = 1. The region marked in green, blue and lilac corresponds to the cytoarchitectonically defined Broca's area with a probability of at least 50% according to [13]. Green: BA 45, blue: BA 44, lilac: intersection of the two. (0.09 MB TIF) [file pone.0005599.s005.tif]

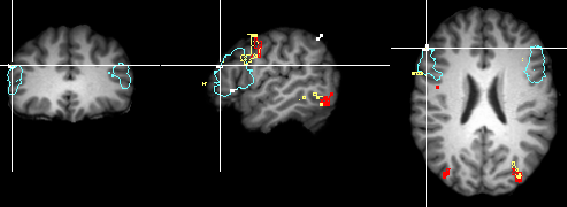

Supplement: Figure S5 — Broca's area is outlined in blue, and regions of activations with Z>3.09, are outlined in white for “hierarchy correct-list correct”, in yellow for “hierarchy correct-baseline” and in red for “list correct-baseline”. The cross hair is placed at (−50, 31, 24) in the Talairach co-ordinate system. Views: coronal y = 31, sagittal x = −50 and axial z = 24. The region marked in blue corresponds to the cytoarchitectonically defined Broca's area (50% according to [13]) (0.09 MB TIF) [file pone.0005599.s006.tif]
