# Supplementary material for: Mathematical Logic in the Human Brain: Syntax
Source: PLoS One. 2009 May 28;4(5):e5599. doi: 10.1371/journal.pone.0005599 (PMC2685028; doi:10.1371/journal.pone.0005599)
Supplement: Table S1 — (0.01 MB DOC) [file pone.0005599.s008.doc]

Table S1

| **AREA** | **Talairach co-ordinates** | | | | | | **Z_max_** |
| --- | --- | --- | --- | --- | --- | --- | --- |
|  | left | | | right | | |  |
| IFG, BA9 | -62 | 10 | 24 | - | - | - | **3.48** |
| MTG, BA22 | -50 | -47 | 3 | - | - | - | **3.46** |
| Sup. parietal lobule, BA7 | -35 | -71 | 48 | - | - | - | **3.56** |
| Sup. parietal lobule, BA7 | - | - | - | 31 | -74 | 45 | **4.31** |
| Precuneus, BA7 | - | - | - | -2 | -65 | 42 | **3.83** |

Activation maxima (uncorrected) of the main effect: “hierarchical (correct+incorrect) vs. list (correct+incorrect)”. Abbreviations: BA: Brodmann area, IFG: inferior frontal gyrus, MTG: middle temporal gyrus.
