# Supplementary material for: Mathematical Logic in the Human Brain: Syntax
Source: PLoS One. 2009 May 28;4(5):e5599. doi: 10.1371/journal.pone.0005599 (PMC2685028; doi:10.1371/journal.pone.0005599)
Supplement: Table S2 — (0.01 MB DOC) [file pone.0005599.s009.doc]

Table S2

| **AREA** | **Talairach co-ordinates** | | | | | | **Z_max_** |
| --- | --- | --- | --- | --- | --- | --- | --- |
|  | left | | | right | | |  |
| Precentral G, BA6 | - | - | - | 40 | -8 | 42 | **3.60** |
| Insula, BA 13 | - | - | - | 37 | 7 | 12 | **3.36** |
| Cuneus, BA18 | -11 | -80 | 18 | - | - | - | **3.67** |
| MFG, BA6 | - | - | - | 22 | 16 | 54 | **-3.48** |
| mFG, BA8 | - | - | - | -2 | 34 | 42 | **-3.63** |
| AG, BA39 | - | - | - | 40 | -62 | 36 | **-3.84** |
| Anterior cingulate, BA32 | -8 | 37 | 24 | - | - | - | **-4.10** |

Activation maxima (uncorrected) for the main effect: “correct (hierarchical+flat) vs. incorrect (hierarchical+flat)”. Abbreviations: AG: angular gyrus, BA: Brodmann area, mFG: medial frontal gyrus, MFG: middle frontal gyrus.
